# Climate Change Adaptation through the Water-Energy-Food Nexus in Southern Africa

**DOI:** 10.3390/ijerph15102306

**Published:** 2018-10-19

**Authors:** Sylvester Mpandeli, Dhesigen Naidoo, Tafadzwanashe Mabhaudhi, Charles Nhemachena, Luxon Nhamo, Stanley Liphadzi, Sithabile Hlahla, Albert T. Modi

**Affiliations:** 1 Water Research Commission of South Africa, 4 Daventry Street, Lynnwood Manor, Pretoria 0081, South Africa; sylvesterm@wrc.org.za (S.M.); Dhesn@wrc.org.za (D.N.); stanleyl@wrc.org.za (S.L.); 2 School of Environmental Sciences, University of Venda, Private Bag X 5050, Thohoyandou 0950, South Africa; 3 School of Agricultural, Earth and Environmental Sciences, University of KwaZulu-Natal, P. Bag X01, Scottsville, Pietermaritzburg 3209, South Africa; Mabhaudhi@ukzn.ac.za (T.M.); hlahlas@ukzn.ac.za (S.H.); modiat@ukzn.ac.za (A.T.M.); 4 International Water Management Institute, Southern Africa (IWMI-SA), 141 Creswell Street, Silverton, Pretoria 0184, South Africa; C.Nhemachena@cgiar.org

**Keywords:** Southern Africa, water-energy-food nexus, climate change, resilience, adaptation

## Abstract

Climate change is a complex and cross-cutting problem that needs an integrated and transformative systems approach to respond to the challenge. Current sectoral approaches to climate change adaptation initiatives often create imbalances and retard sustainable development. Regional and international literature on climate change adaptation opportunities and challenges applicable to southern Africa from a water-energy-food (WEF) nexus perspective was reviewed. Specifically, this review highlights climate change impacts on water, energy, and food resources in southern Africa, while exploring mitigation and adaptation opportunities. The review further recommends strategies to develop cross-sectoral sustainable measures aimed at building resilient communities. Regional WEF nexus related institutions and legal frameworks were also reviewed to relate the WEF nexus to policy. Southern Africa is witnessing an increased frequency and intensity in climate change-associated extreme weather events, causing water, food, and energy insecurity. A projected reduction of 20% in annual rainfall by 2080 in southern Africa will only increase the regional socio-economic challenges. This is exacerbating regional resource scarcities and vulnerabilities. It will also have direct and indirect impacts on nutrition, human well-being, and health. Reduced agricultural production, lack of access to clean water, sanitation, and clean, sustainable energy are the major areas of concern. The region is already experiencing an upsurge of vector borne diseases (malaria and dengue fever), and water and food-borne diseases (cholera and diarrhoea). What is clear is that climate change impacts are cross-sectoral and multidimensional, and therefore require cross-sectoral mitigation and adaptation approaches. In this regard, a well-coordinated and integrated WEF nexus approach offers opportunities to build resilient systems, harmonise interventions, and mitigate trade-offs and hence improve sustainability. This would be achieved through greater resource mobilisation and coordination, policy convergence across sectors, and targeting nexus points in the landscape. The WEF nexus approach has potential to increase the resilience of marginalised communities in southern Africa by contributing towards attaining the Sustainable Development Goals (SDGs 1, 2, 3, 6, 7, and 13).

## 1. Introduction

Climate change-associated extreme weather events, such as droughts and flooding, have emerged as the biggest challenges faced by the fast growing and emerging economies in southern Africa. Temperature is expected to continue warming and rainfall patterns are projected to continue changing, thereby increasing risk and uncertainty in a region with low adaptive capacity [1]. Changes in temperature and rainfall will be felt variably across the region, with some areas becoming warmer and wetter, while others will become warmer and drier [2]. Previous studies on climate change scenarios highlight that the latter scenario will be more dominant across southern Africa, with climate change impacts being mostly associated with declining rainfall [2,3,4]. In terms of economic development, climate change is described as a wicked problem, because it is complex and straddles various sectors and levels [5]. Responses to climate change therefore necessitate transdisciplinary or cross-sectoral approaches [6]. Current approaches to climate change adaptation are similar to the region’s sectoral framing for planning and development, which has often led to mal-adaptation as trade-offs are not accounted for.

Within the region, the dominant development paradigm is underpinned by sectors that are highly exposed to climate variability and change, especially agriculture, hydropower, and water infrastructure [7,8]. The sectoral approach has been cited as another obstacle to sustainable development and efficient resource utilisation [6,9]. As such, there is a need to approach development from a cross-sectoral perspective as the challenges faced are cross-cutting and most resources are also transboundary in nature [2,9]. In this regard, emerging cross-sectoral approaches such as the water-energy-food (WEF) nexus could be useful [10]. The WEF nexus is broadly defined as an approach that considers the interactions, synergies, harmonisation, and trade-offs between water, energy, and food when undertaking the management of these resources [9]. It is because of this cross-sectoral approach to resource planning and management that the WEF nexus is an important tool in climate change adaptation. WEF resources are inextricably linked, with usage within one sector influencing the use and availability in the adjacent sectors. The WEF nexus has been studied widely, with various studies focusing their analyses on niches or sectors, be they political, social, or scientific perspectives [11]. The WEF nexus tackles these ‘silo’ approaches to resource management. Unlike Integrated Water Resource Management (IWRM), which is water-centric in nature, the WEF nexus considers all resources with equal weighting and manages resources more holistically through a multi-centric philosophy [6,12].

It is anticipated that by 2050, water and food demands will have increased by over 50%, while the global demand for energy would have nearly doubled, leading to competing needs for limited resources [13]. These challenges would exacerbate a host of other challenges such as malnutrition, poor health and sanitation as well as migration [14,15]. The increasing body of evidence on climate change impacts, as well as increasing demand from a growing population, requires a cross-sectoral approach such as the WEF nexus, to promote sustainable development [6]. The WEF nexus provides a framework for managing synergies and trade-offs between water, energy, and food in the context of emerging constraints on sustainable development. The WEF nexus, as a socio-ecological systems approach, provides an opportunity to sustainably address complex problems such as climate change adaptation [9], while still promoting regional economic development.

Currently, national and regional development targets remain elusive due, in part, to sectoral approaches to resource management, which inadvertently create imbalances and inefficiencies in resource allocation and utilisation, respectively [9]. Dependence on climate sensitive WEF resources in southern Africa worsens vulnerabilities. Consequently, the region is classified as a climate change hot spot [16]. This is particularly concerning for the Southern African Development Community (SADC) region due to the recurrence of climate change associated extreme weather events and high climatic variability, particularly droughts and floods. Water, energy, and food resources are vital for human wellbeing, poverty reduction, and sustainable development and their management is vital to achieving the Sustainable Development Goals (SDGs) [17]. The WEF nexus approach offers opportunities to integrate climate change adaption strategies [18]. This would translate to savings from costs associated with duplication of developmental projects, increased efficiencies due to streamlining of activities, and higher likelihood of success due to mitigation of trade-offs and amplification of synergies [6]. Adoption of the WEF nexus approach calls for transformative change with regards to institutional arrangements, governance, and alignment as well as public and private participation. The pressure to produce more food and energy with less water requires a paradigm shift from the current ‘silo’ approach to cross-sectoral approach through the WEF nexus [6]. Continuing with the business as usual approach would cause socio-economic insecurities due to increased demand of essential resources to satisfy the needs of a growing population.

While it is acknowledged that climate change poses the single greatest threat to regional growth and development, most climate change adaptation strategies and plans are still formulated and implemented along sectoral lines [6]. This tends to negate the complexity of climate change and the need for a systems approach in developing adaptation plans. The absence of a cross-sectoral approach to climate change adaptation risks mal-adaptation. The objective of the review was to highlight the impacts of climate change on water, energy, and agriculture sectors in the SADC region, and explore opportunities for the WEF nexus in developing cross-sectoral sustainable climate change mitigation and adaptation strategies and plans. In the review, agriculture and land are a proxies of food in the WEF context. Scientific articles, published reports, and accessible grey literature related to climate change and WEF nexus were reviewed, focusing on the SADC region.

## 2. The Study Area

**Figure 1:**
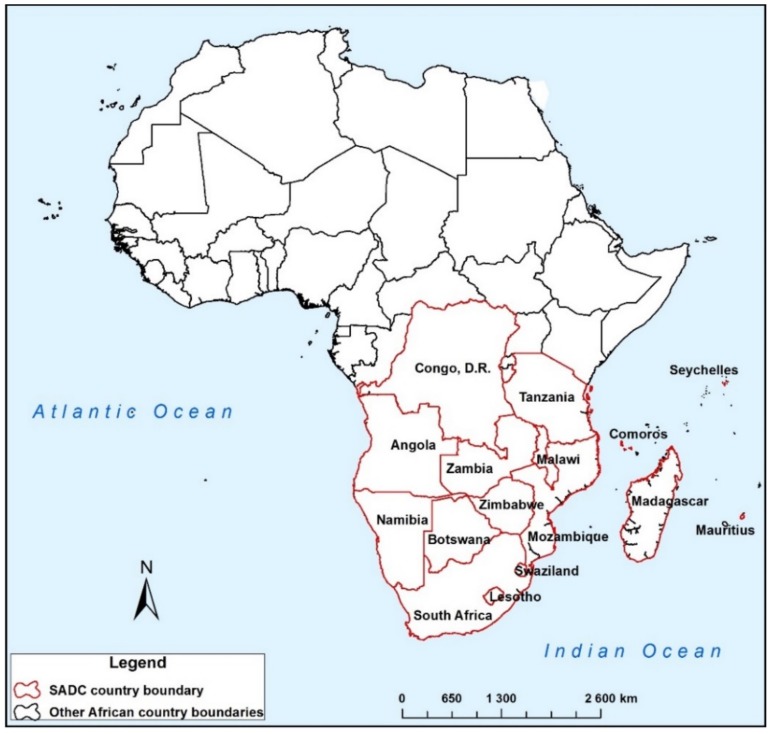
Location of Southern African Development Community (SADC) countries in Africa.

The SADC is a regional economic community comprising 15 southern African states, mainly Angola, Botswana, Comoros, Democratic Republic of Congo, Swaziland, Lesotho, Madagascar, Malawi, Mauritius, Mozambique, Namibia, Seychelles, South Africa, Tanzania, Zambia, and Zimbabwe (Figure 1). The region has a combined area of 986,246,000 ha, of which only 6.11% is cultivated. Seventy five percent of the region is either arid or semi-arid, with a highly variable and uneven climate through the region, varying from desert, through temperate, savannah, and equatorial. Rainfall varies between 650 mm in the driest areas and 2000 mm in the wettest regions [19]. Agriculture is the largest sector employing about 70% of the working class, yet it is mainly rainfed [20]. Agriculture alone sustains the livelihoods of over 60% of the population. Land with irrigation potential is approximately 20 million ha, of which only 3.9 million ha is equipped for irrigation, accounting for about 6.6% of cultivated area [20]. The region is endowed with vast but unexploited energy resources [21]. The resource rich 15 transboundary river basins of the region are an opportunity for regional integration and cooperation.

## 3. Climate Change Impacts on WEF Resources in the SADC Region

### 3.1. Climate Change Impacts on Water Resources

**Figure 2:**
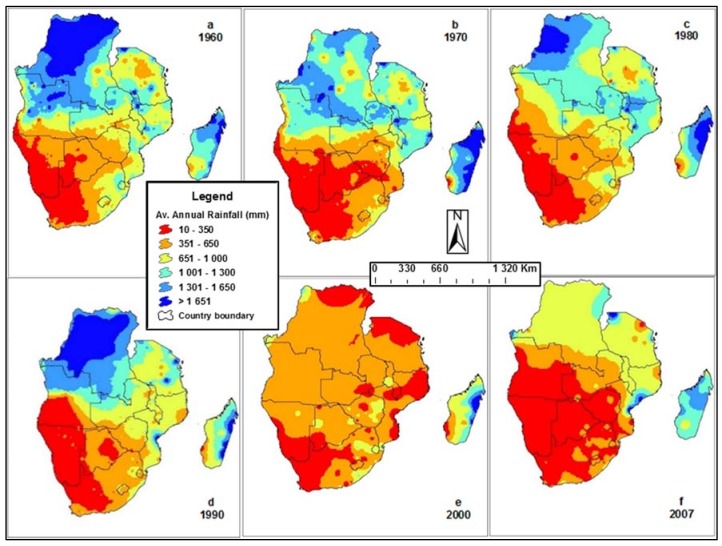
Spatio-temporal changes in annual rainfall distribution and variability in the SADC region in 1960, 1970, 1980, 1990, 2000 and 2007. Source: Nhamo et al. [2].

Figure 2 shows rainfall variability and how it has been decreasing over the years from 1960 to 2007. The region is marked by a great spatio-temporal variability in climate and water resources, particularly in southern drier countries [2]. Figure 2f represents the rainfall pattern of 2007, showing that almost half of the surface area of the SADC region has become arid, receiving less than 650 mm of rainfall. It is projected that for southern Africa, climate change impacts would mostly be felt through water resources [3]. The estimated reductions of about 20% in rainfall by 2080 could exacerbate water, energy, and food insecurity if no action is taken [16]. This would negatively impact food production and energy generation. In addition, there would be indirect impacts on nutrition, health and sanitation, water conflicts, among other social challenges.

**Figure 3:**
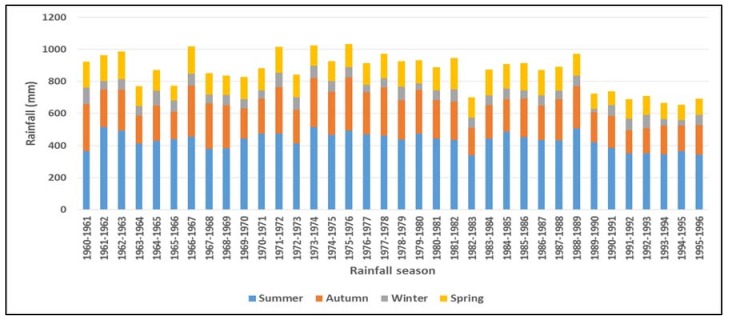
Historical changes in seasonal rainfall in the SADC region (1960 to 1996). Source: Nhamo et al. [2].

Figure 3 shows the annual average rainfall by season (summer, autumn, winter, and spring) from 1960 to 1996 in the SADC region. In southern Africa the summer season (December to February) is the wettest and, winter (June to August) is the driest. Autumn and spring are transitional seasons ushering into winter and summer, respectively [22]. For the period under review, rainfall was highly variable from 1960–1961 to 1988–1989 but from 1989–1990 onwards the variability was accompanied by a continued decrease in rainfall, particularly during the summer (Figure 3).

Reductions in annual rainfall and the unreliability of surface water resources have resulted in an increase in the use of groundwater resources, including in urban areas [6]. Urban households in some countries in southern Africa have resorted to digging wells as domestic taps usually run dry [8]. Although groundwater is often preferred for domestic use because of its generally good microbial quality in its natural state, it can easily be contaminated if protective measures at the point of abstraction are not implemented and well-maintained. Some groundwater can naturally contain fluoride and arsenic, elements that can cause health issues to people [15]. The quality of groundwater sources from shallow wells in urban areas of the region is often very low due to poor waste management and source protection, which poses a health risk to users [15]. As demand for groundwater is anticipated to increase in southern Africa due to population growth and changes in future climate, there is need for a detailed knowledge of both water quantity and quality so as to use the resource sustainably.

Reduced annual rainfall impacts energy generation and food production, compromising regional security and exacerbating regional vulnerabilities [23]. For example, the 2015/16 El Niño Southern Oscillation (ENSO) induced drought affected the whole region, resulting in more than 40 million people (14% of the SADC population) to be food insecure [23]. The drought caused 643,000 livestock deaths and an overall maize deficit of 5.1 million tonnes [23]. Dam water levels declined, causing energy insecurity in some countries that depend on hydro-power for their electricity [6]. The energy insecurities in the region are exacerbated by population and industrial growth as well as improving standards of living and changing diets, factors that increase the demand of resources like water, energy, and food [6].

### 3.2. Climate Change Impacts on Agriculture

**Figure 4:**
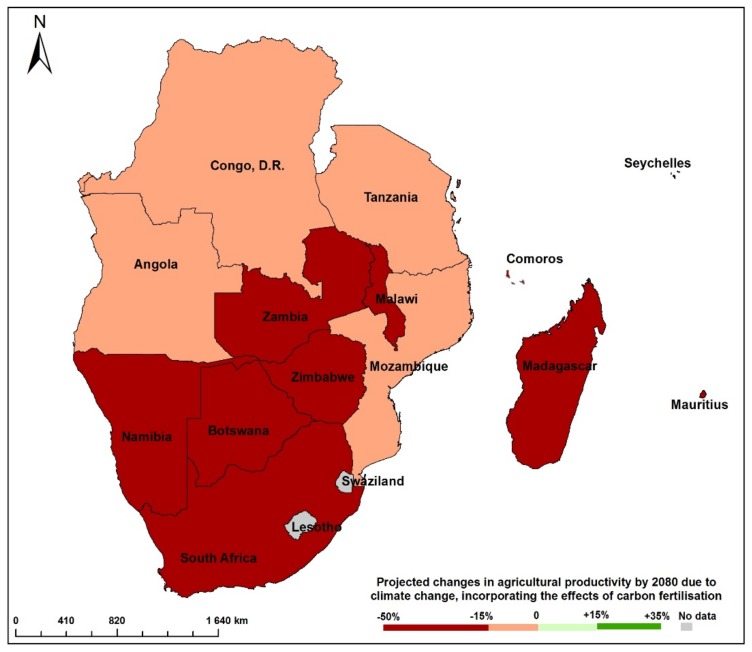
Projected changes in agricultural productivity in 2080 due to climate change. Source: Adapted from Ahlenius and UNEP/GRID-Arendal [26].

Changes in climate are expected to severely affect the performance of the agriculture sector, with detrimental effects on progress in achieving food and nutrition security [24,25]. The Fourth and Fifth Assessment reports (AR4 and AR5) reported with medium confidence that agricultural systems in Africa (including southern Africa) were vulnerable to climate change impacts. Empirical evidence shows that agricultural productivity in SADC would be significantly affected by projected changes in climate. For example, Figure 4 shows that agricultural productivity in the region will decrease from 15% to 50% by 2080 due to climate change [26]. This is worrying as the region, with the exception of South Africa and Zambia, already faces deficits for the major staple crops. Any further decreases would worsen regional food and nutrition insecurity.

**Figure 5:**
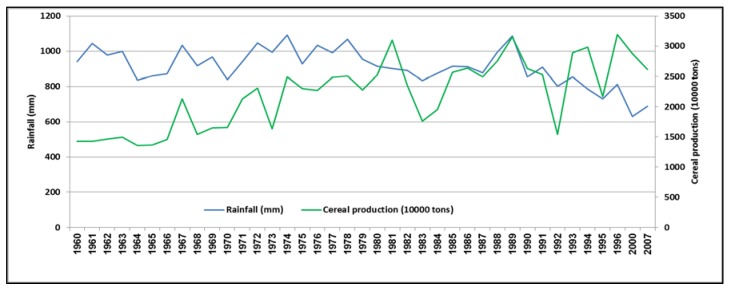
Relationship between rainfall and cereal production in the SADC region from 1960 to 2007. Source: Developed by authors from ReSAKSS data.

Figure 5 shows the relationship between rainfall and cereal production in the SADC region from 1960 to 2007. For the period under review, rainfall was observed to be highly variable. Similar to Figure 3, a drastic decline was observed from 1987 onwards. Currently, average regional rainfall is less than 800 mm. Cereal production has generally followed the trend in rainfall variability, but with an increasing trend. Although cereal production has significantly increased along the years, this is because of the increase in irrigated area in the region. However, as water resources become scarcer, future increases in the yields of cereal staples will have to come from improvements in water productivity, as opposed to increasing water withdrawals. Cereal production has been increasing simultaneously with population, thus agriculture has been failing to meet the food requirements of the growing population [27,28].

Climate change is envisaged to contribute substantially to food insecurity in the future through reductions in food production and subsequent food prices increases [28]. Climate mitigation efforts will result in high energy usage and may cause increases in food prices [1]. Water shortages due to recurring droughts and increased crop water use will exacerbate the challenges of water, energy, and food insecurity. Some lands may become climatically unsuitable for agriculture, causing more competition for land [1].

The food system is a major source of greenhouse gas emissions, and future intensification of agriculture to compensate for reduced yields and productivity, together with the anticipated increased demand for animal-sourced products due to increasing affluence, could further increase these emissions [1]. Current approaches to climate change adaptation mostly focus on improving access to water and increasing area under irrigation. However, there is a need to debate the trade-offs with energy and water as well as balancing demands from other strategic sectors. Regional food and nutrition security priorities should not outweigh such considerations as that would render any such initiatives unsustainable. In this regard, the WEF nexus could offer opportunities for sustainably addressing food and nutrition security while mitigating trade-offs with water and energy.

### 3.3. Climate Change Impacts on the Energy Sector

**Figure 6:**
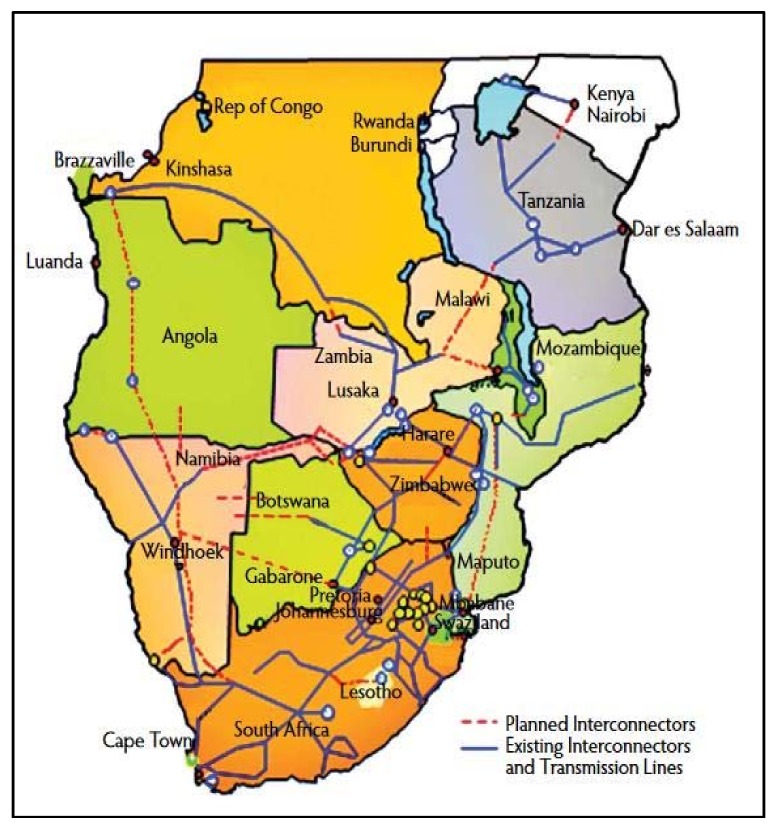
Distribution of energy infrastructure in shared watercourses in the SADC. Source: SAPP [41].

The SADC region is endowed with vast energy resources, namely hydropower, coal, biomass and solar (Figure 6), although availability varies from country to country [29]. However, only 24% of the total population in the region and 5% of rural population have access to electricity [6]. Seventy-five percent of electricity generation is from coal, a source of carbon dioxide, which causes greenhouse gas [6,29]. The overall hydropower potential of the SADC is estimated at approximately 1080 terawatt hours per year (TWh/year), yet the current utilized capacity is less than 31 TWh/year [21]. The untapped hydropower generation potential in regional states such as Angola, the DRC, Mozambique and Zambia (countries with reliable water resources) has capacity to supply the whole region with electricity [29,30]. The shared power grids whose electricity is generated from shared watercourses (Figure 6) is an an opportunity for regional intergration and cooperation.

Households without electricity tend to rely on biomass such as wood and charcoal to meet their energy needs [31,32]. However, if people harvest the wood at a faster rate than trees can grow, the capacity of natural forests to contribute towards climate change mitigation will be diminished [9]. Trees are described as carbon sinks as they absorb carbon which causes greenhouse gasses [32]. Deforestation and desertification also have implications on water resources and food production due to erosion, and declining arable lands, respectively. Soil erosion can result in the transportation of top soil and sediments, which are rich in organic materials, nutrients, chemicals, and soil life, into water bodies, causing river siltation, water pollution, and in some instances, water eutrophication, which threaten aquatic life [24]. The loss of rich soil also affects food production by removing soil nutrients and degrading arable land [24].

Lack of investment and financing have impeded regional efforts to tap into its abundant clean and renewable energy resources (wind and solar). Together with hydropower, fossil fuel-based energy sources are expensive, yet they are the largest sources of electricity for most SADC countries [33]. The push for alternative sources of energy is not only caused by the growing energy demand, but the use of fossil fuels and biomass has been found to be environmentally unfriendly, contributing the most to greenhouse gas emissions and are the main causes of climate change [34]. Human-induced climate change is the cause of the recurrence of climate change associated extreme weather events such as storms, droughts, and floods [2].

As the demand for energy continues to increase due to population and industrial growth and urbanisation, energy insecurity has worsened within the region, causing frequent power blackouts in most countries. The worsening energy insecurity also threatens the region’s economic development agenda. For example, in 2008–2009, the average electricity outage was 6.70 h with corresponding losses of 5.4% of annual sales [35]. According to the SADC Regional Infrastructure Development Master Plan (RIDMP) of 2012, assuming an average economic growth rate of 8% per annum, energy demand is expected to increase to more than 77,000 MW by 2020 and to over 115,000 MW by 2030, exerting more pressure on water resources [36]. The increasing demand for energy to drive industrialisation and job creation has naturally led to prioritisation of energy development, but unfortunately leaning toward the conventional high carbon and water intensive energy options. This has created conflicts with water and food sectors. For example, in South Africa, coal mine expansion for power generation has already come into conflict due to competition for prime arable land and pollution of ground and surface water. Such conflicts may escalate under climate change. As such, similar to the need to achieve food and nutrition security, the drive for energy security should consider trade-offs with the water and food sectors. This provides more impetus for adopting a WEF nexus approach to regional economic development and climate change adaptation planning.

### 3.4. Implications for Nutrition, Human Health and Wellbeing

Climate change affects human health and wellbeing both directly and indirectly. For example, heatwaves have increased mortality rates, while flooding and droughts have reduced agricultural production [37,38]. Heatwaves have been responsible for numerous deaths and illnesses in older age and low income groups, as well as those with pre-existing cardio-respiratory diseases [39]. In 2015, for example, India experienced what is now called the fifth deadliest heatwave in history, killing over 2000 people after temperatures soared to over 45 °C [40]. Githeko and Woodward [39] noted that heatwaves were projected to increase in mid to high latitude cities, and would mostly affect populations who were unable to adapt and had limited access to air conditioning.

Climate change also has an impact on mental health and psychological and social wellbeing. It can cause minimal stress and distress symptoms, and clinical disorders, such as anxiety, depression, post-traumatic stress disorder (PTSD), recovery fatigue, substance abuse, and suicidal tendencies [41,42]. Previous studies noted that the threat of climate change is a psychological and emotional stressor as people become affected by direct exposure to climate-related events and information about the phenomenon and its effects [40,41,42,43]. Low income groups, indigenous groups, children, old people, women, outdoor labourers, and people with pre-existing health conditions, tend to be disproportionately impacted, mentally and physically, by the impacts of climate change [43,44]. The hardest hit are the marginalised who already spend a disproportionate amount of resources on food [44]. The United Nations Development Programme (UNDP) projects an increase in food prices by 30–50% in the coming decades due to adverse environmental factors, namely, land degradation, climate change, and water scarcity; low-income groups will be the hardest hit as such increases bear health implications [45]. Niang et al. reported that, in 2009, global food price increases contributed to the deaths of approximately 30,000 to 50,000 malnourished children in sub-Saharan Africa [1]. Thus, climate change is increasing the problems of undernutrition due to low yields and increases in food prices. These challenges are threatening to reverse development gains and impede the achievement of SDGs on no poverty, zero hunger, good health and well-being, good water and sanitation, affordable and clean energy, and climate action (Goals 1, 2, 3, 6, 7, and 13).

Apart from its impacts on water, energy, and agriculture, climate change is also projected to increase health risks, particularly on already vulnerable populations [46,47]. Some groups of people are highly susceptible to climate sensitive health impacts because of age (children and elderly), gender (pregnant women), social marginalisation (associated in some areas with indigenous populations, poverty or migration status), among others health issues [47]. Infectious diseases, such as water-borne diseases, are highly sensitive to prevailing weather conditions. Transmission and geographical range of diseases like malaria is expected to increase due to climate change [46]. Apart from these health challenges, climate change will be associated with new and emerging health issues related to heatwaves and other extreme climate events. About 22.5 million people are displaced by climate or weather-related disasters annually, and the number is projected to increase in the short-term [48]. Also, food and water insecurity bring about the challenges of malnutrition and undernutrition.

## 4. Climate Change Adaptation in the Context of the WEF Nexus

As already alluded to, climate change is already affecting WEF resources in the SADC and exerting further pressure on already scarce resources. The challenge requires innovative and transdisciplinary approaches on mitigation and adaptation strategies that enhance resilience while concurrently improving livelihoods [28].

Climate change adaptation through an integrated and cross-sectoral management of resources remains important for regional economic development and improving the livelihoods of people [49,50,51]. Responses to climate change range from autonomous coping strategies to reactive interventions towards climate variability and extreme weather events, and proactive interventions to long-term changes in climate. Reactive/autonomous adaptations include changes in production and management practices (such as changes in crop mixes, and crop varieties) in response to changes in local climatic and growing conditions. Proactive interventions, on the other hand, involve planned policy and investment decisions to enhance adaptive capacity of target agricultural systems, such as investments in efficient irrigation systems and new crop varieties [50,52,53]. While reactive/autonomous responses are useful in the short-term, it is proactive interventions that will contribute to long-term adaptation and sustainability. In that regard, it is important for the region to plan and implement these interventions in a sustainable manner that contributes to resilience building.

The SADC region needs to adopt water management practices and aim to produce more food and energy with less water resources. One such initiative would be the promotion and cultivation of indigenous underutilised crops that suit local harsh environmental conditions [54]. Cultivating indigenous crops would allow for agricultural expansion, and marginal areas currently deemed unsuitable for crop production without increasing water withdrawals for agriculture which currently stands at 70% of available water resources [55]. Their low input requirements give them an economic advantage over adapted crops like maize, rice, and wheat [56]. Indigenous underutilised crops help to ensure food and nutrition security as part of a balanced diet, when adapted crops fail, or in between harvests [57]. In addition, this could contribute to utilising currently underutilised large tracts of land that have been deemed unsuitable for the production of major staple commercial crops. Thus, their cultivation would neither create new demands for water nor would they compete for current prime agricultural land. Further improvements to water and energy use efficiencies related to irrigated agriculture would also mitigate the risk of increasing water and associated energy demands from agriculture. In this regard, promotion of micro irrigation, use of solar powered pumps and other forms of farming, such as hydroponics, would go some way in mitigating trade-offs associated with increasing food production. Concurrently, research and development should focus on multi-purpose agro-systems which deliver on more than just food but also water and energy. For example, the development of multi-purpose dams that serve irrigation, power generation, aquaculture, and eco-tourism.

From an energy perspective, the adoption of cleaner and renewable sources of energy would result in saving water and that would also ensure energy security in a region that depends on hydro and coal energy sources. Hydropower is climate sensitive and any negative changes in rainfall regimes significantly impact energy availability. On the other hand, fossil energy is not environmentally sustainable and is the main contributor of greenhouse gas emissions. Southern Africa can take advantage of its abundant clean sources of energy such as solar and wind [58]. Harnessing the abundant solar and wind energy in the region would not only result in energy security but would also result in huge water savings. Energy deficits in southern Africa have effectively stunted regional development, as industries always struggle in most regional countries as electricity remains costly and inconsistent [21]. Also, 70% of the population do not have access to reliable energy [21]. The use of renewable sources of energy (wind and solar) is a better option capable of sustainably supplying enough energy for the region. They also offer advantages of employment creation, proximity to point-of-use and, in many cases, less reliance on concentrated sources of energy.

## 5. Integrating Water, Energy and Agricultural Sectors in Climate Change Plans

Climate change exacerbates the problems of essential resources insecurity and vulnerability in southern Africa as WEF resources are climate sensitive. In most cases, these challenges derail development targets [9]. Cross-sectoral management of WEF resources is vital to achieving the SDGs as the WEF nexus approach is designed to integrate resource development in a coordinated manner and it’s a pathway to climate change adaptation [17]. Such coordination translates to savings from costs associated with duplication, increased efficiencies due to streamlining of activities, and higher likelihood of success due to consideration of WEF nexus trade-offs and synergies [18]. As climate change will have a multiplier effect on several stressors (growing population, urbanisation, migration, and industrial growth), the WEF nexus could be a pathway towards balancing competing demands for resources [11,59].

**Figure 7:**
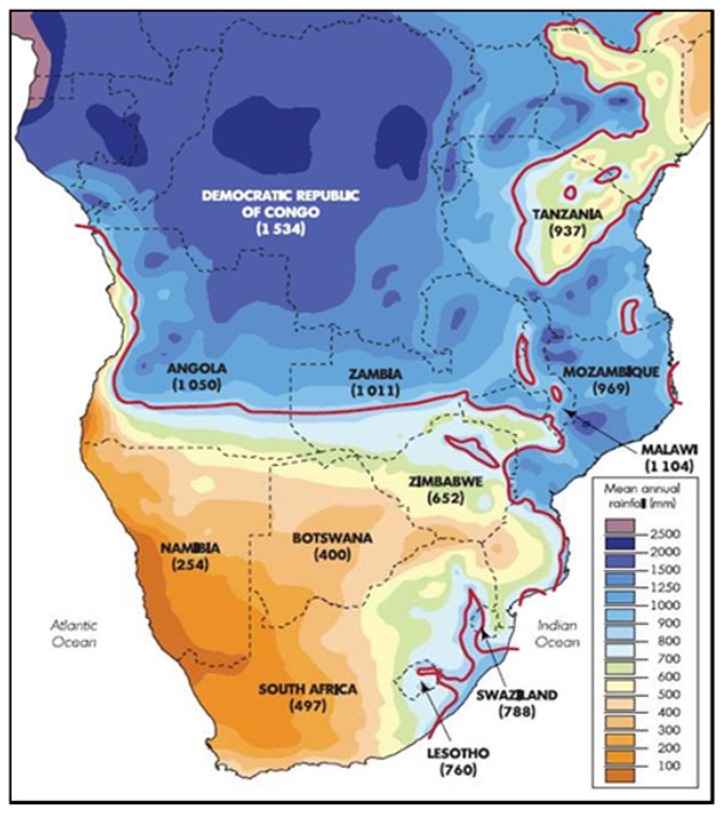
Spatial distribution of mean rainfall over southern Africa. Source Davis [60].

Hydrological resources of the SADC region are unevenly distributed (Figure 7) [22]. Seventy-five percent (75%) of the region is water scarce (physical and economic) due to the uneven distribution of water resources [2]. Mean annual runoff volume is 650 km^3^, a low volume for a region that depends on rainfed agriculture and hydro-power for energy [55]. The uneven distribution of hydrological resources favours a coordinated approach with benefit-sharing mechanisms to manage the region’s resources. The region could also benefit from water transfer and hydropower generation as the northern parts of the region have abundant water resources and could supply the southern parts with water [29]. The shared natural resources provide a basis for the development of regional instruments to support cooperation in resource management for inclusive development and cement regional integration, given that the impacts of and responses to climate change are generally cross-sectoral [6]. The integration of climate change into regional policies, plans, and strategies is an important means of encouraging action on climate change adaptation, as policy gives political will.

### 5.1. Current SADC WEF Nexus Initiatives

**Figure 8:**
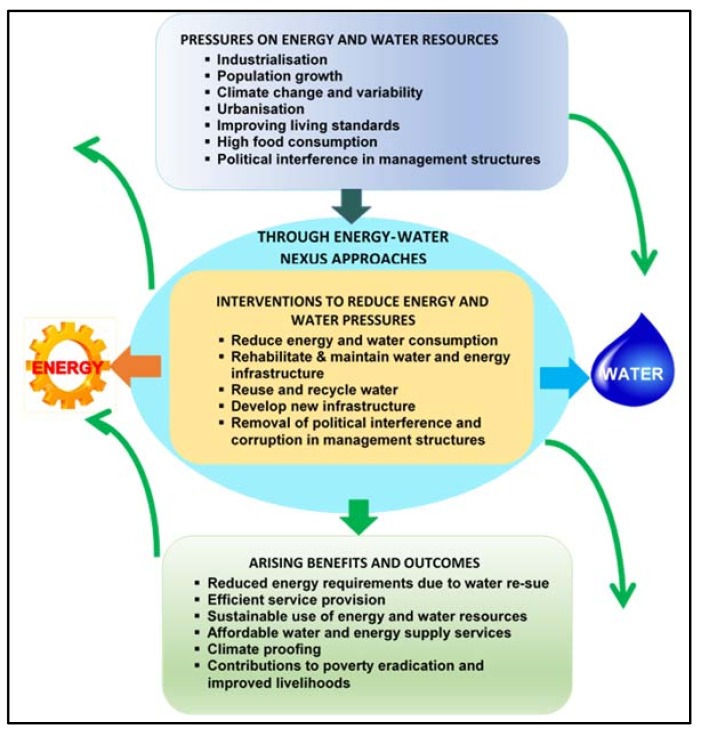
Energy-Water Action Plan conceptual framework. Source: Adapted from The World Bank [27].

The SADC region produced the WEF Nexus Action Plan, which is incorporated in the RSAP IV, in realisation of the importance of the nexus in regional socio-economic security and cooperation and integration and due to the need to respond to the recurrence of drought in the region [60]. The aim of the WEF Nexus Action Plan (Figure 8) is to create an enabling environment for accelerated industrial growth and pilot the nexus to facilitate better understanding of the nexus benefits. The action plan recognises the role of the nexus in adapting to the challenges posed by population growth and climate variability and change, as well as in optimising resource use in order to achieve regional goals and targets. The plan calls for a regional nexus assessment study to provide policy recommendations as well as strategic actions that are informed by research and scientific evidence. Key activities include mobilising resources for a regional nexus study in collaboration with other sectors and identifying and implementing regional nexus demonstration projects and studies.

Despite the presence of the WEF Nexus Action Plan, there is little or no evidence of cross-sectoral linkages in institutions, policies and current projects. Some activities in recognition of the importance of the nexus include the SADC multi-stakeholders water dialogue and that focus on exploring nexus opportunities in providing coherent and well-planned development and use of resources. The multi-stakeholder dialogues also focus on regional value chains and job creation through the WEF nexus. While these efforts are commendable, there is a need to start developing action plans with clear timelines on how the WEF nexus initiatives and projects.

### 5.2. Strengthening WEF Nexus Related Institutions and Policies in Southern Africa

The SADC Treaty is the overarching framework for the region, whose objective is to achieve economic development, peace and security, and growth; and also, to alleviate poverty and improve the livelihoods of the people, all of which are achieved through regional integration [61]. The SADC region has ratified several legal frameworks and established institutions related to the WEF nexus [6]. However, there has been lack of coordination among policies and institutions, and that has resulted in policy spill-overs and transferring challenges to other sectors [62]. There is, therefore, a need for policy harmonisation, which is the very reason of the WEF nexus approach in resources management. To-date, the region has ratified the following WEF nexus-related institutions and policies:

(a) The Regional Strategic Action Plan IV (RSAP IV) [60], based on the SADC Water Policy and Strategy, which advocates for equitable and sustainable utilisation of water for social and environmental justice, regional integration, and economic benefit for present and future generations. The RSAP IV emphasises infrastructure development and water resource management for food security in the water-food nexus, and an urgency to act in light of climate variability and change.
(b) The SADC protocol on shared watercourses [63] promotes co-operation for judicious, sustainable, and coordinated management, protection, and utilisation of shared watercourses, and advancement of SADC’s agenda on regional integration and poverty alleviation. Most shared river basins have basin level agreements in place, which oversee the management of the basins. Examples of shared river basin agreements include the Limpopo Watercourse Commission (LIMCOM), Okavango River Basin Commission (OKACOM), Orange-Senqu River Commission (ORASECOM), and Zambezi River Basin Commission (ZAMCOM).
(c) The Southern African Power Pool (SAPP) [64] highlights the development and updating of a regional electricity master plan, and the development and utilisation of electricity in an environmentally sound manner, whilst emphasising the need for universal access to affordable and quality services. The SAPP is guided by the Protocol on Energy and enhances regional co-operation in power development and trade, and to provide non-binding regional master plans to guide electricity generation and transmission infrastructure delivery.
(d) The SADC’s Renewable Energy and Energy Efficiency Strategy and Action Plan (REEESAP 2016–2030) [65] envisions fostering a regional coherence towards developing renewable energy and energy efficient technologies and services by 2030. The SADC Centre for Renewable Energy and Energy Efficiency (SACREEE) is the regional institution mandated to implement the REEESAP, harmonise and coordinate efforts, act as a regional renewable energy and energy efficiency promotion agency and a knowledge hub.
(e) The SADC Regional Agricultural Policy (RAP) [20] envisions integrated approaches on water resources management, emphasising on improving agriculture performance to meet regional food and water security, as well as attaining sustainable economic development objectives at a regional level. The SADC’s Regional Indicative Strategic Development Plan (RISDP) [66] derived from the Africa-wide Comprehensive Africa Agricultural Development Programme (CAADP), aims to increase double irrigated area from 3.5 to 7% by 2025 [69].
(f) The WEF Nexus Action Plan [67] recognises the role of the nexus in adapting to the challenges posed by population growth and climate variability and change, as well as in optimising resource use to achieve regional goals and targets.
(g) The SADC secretariat produced a policy paper on climate change which emphasises a cross-sectoral approach to mitigate climate change impacts. The policy paper highlights two key aspects to the future SADC climate change programme; (a) to establish an implementation strategy and (b) to develop an action plan.

## 6. Recommendations Based on the WEF Nexus Approach

The primary goal for the SADC is to foster regional growth and integration which is premised on the realisation that the SADC region is unified by a common history, culture, transboundary agreements, and shared natural resources as highlighted in the SADC Treaty [61]. At the country level, countries have similar goals on poverty alleviation, improving the quality of life for its inhabitants, economic development and job creation as also stated in the SADC Treaty. Regional countries also face similar challenges such as increasing population, increasing rural–urban migration, food insecurity, unemployment, and inequality. Water, energy, and food security are central to the region’s plans for sustainable economic development and transformation. It is in this regard that the WEF nexus offers significant opportunities for a coordinated approach to addressing some of the region’s pressing challenges and achieving regional goals under climate change.

Research has proposed several climate change adaptation strategies for the region, which include; (i) promoting climate smart agriculture; (ii) developing Early Warning Systems (EWS); (iii) integrated water resources management; (iv) promoting renewable energies with low carbon footprint; and (v) increasing monitoring and modelling capacities across each of the WEF sectors [68]. However, these approaches are either water, energy, or food-centric and driven by the individual sectors. As already alluded to, this risks causing mal-adaptation through creating imbalances. Based on the WEF nexus approach and the vision of the SADC Treaty, as well as achieving related SDSs, we recommend the following for developing climate change adaptation strategies and plans:

■ ***Regional integration***. Agro-ecological zones transcend political boundaries and sovereignty. The WEF nexus provides a meaningful platform for coordinated access, utilisation, and beneficiation of these resources and potential for effective synergies and trade-offs between the WEF nexus components at regional level [69,70]. The WEF nexus also provides an opportunity to harmonise existing institutions and policies and translate them into coordinated balanced strategies that can contribute towards inclusive development, socio-economic security, and regional integration.
■ ***Sustainable economic development***. The WEF nexus has become central to regional dialogues on economic development and subsequent monitoring of the SDGs. This is because the WEF nexus promotes the inseparable linkages between the use of resources to provide basic and universal rights to food, water, and energy security [51]. Adoption of the WEF nexus in the SADC region is envisaged to benefit sustainable resource use that will promote sustainable and inclusive economic development, job creation, and improving the livelihoods of people. This recommendation is well aligned to SDGs 1, 2, and 7 on no poverty, zero hunger, and affordable and clean energy, respectively.
■ ***Eradicate poverty and Improved human well-being***. Owing to historical imbalances, most of the SADC’s population (~60%) is afflicted by poverty and reside in rural areas. They still lack access to clean and safe drinking water, sanitation, energy, and face chronic food insecurity due to reliance on rainfed agriculture. Consequently, much of the region’s policies have been driven by the need to improve human well-being through improved service delivery. Human well-being is at the core of the WEF nexus. The WEF nexus, through coordinated and shared resource utilisation, has potential to improve human livelihoods. For example, of the 2300 km^3^/annum of available renewable freshwater water resources, a meagre 44 km^3^ is abstracted and 14% is stored [29]. The balance of this water, which could be captured and redistributed to drier parts, currently either flows to the ocean or evaporates, i.e., non-beneficial. Implementing a regional WEF nexus plan could unlock these water resources and benefit the drier southernmost countries of the SADC region. The WEF nexus could also assist in sustainably utilising the 50 million ha of irrigable land (currently only 7% is irrigated) as well as increasing energy generation through harnessing the hydropower potential (150 GW potential versus 12 GW actual) [26] through utilising the region’s underutilised dams. These projects, and others, have potential to improve water, energy and food security in the region thus, contributing to improved human well-being. The recommendation on poverty eradication and improved human well-being is aligned with SDGs 1, 2, 3, 6, and 7 on no poverty, zero hunger, good health and well-being, clean water and sanitation, and affordable and clean energy, respectively.
■ ***Harmonisation of institutions and policies***. A lot needs to be done to unlock the potential of the WEF nexus approach to effectively exploit the many interlinked development opportunities within the SADC region. It is only recently that the WEF nexus has found its place in regional policy formulation as most existing instruments were developed without adequate consideration for cross-sectoral synergies and trade-offs. The lack of vertical and horizontal linkages between sectoral institutions has created an imbalance and duplication among the sectors in terms of demand and supply. The cross-sectoral efforts have remained static, such as considering water for food or energy for food, disadvantaging other sectors. While the SADC’s RAP has contributed to an increase in food production in the region, it has resulted in huge pressure on water and energy resources and has weakened the sustainability of agriculture. The WEF nexus promotes an integrated approach to resource use, thus promoting cross-sectoral balance and inclusive development. Harmonising institutions and policies among the three sectors minimises cross-sectoral conflicts, maximises synergies, mitigates trade-offs, reduces implementation costs, and achieves policy objectives through a systems approach. Harmonised polices ensure systematic promotion of mutually reinforcing strategies and instruments and resolve policy conflicts in order to meet the competing demands for resources. For example, the RISDP provides an opportunity for joint planning and implementation of the WEF nexus to maximise synergies among the WEF sectors. This could lead to improved hydropower development and irrigation expansion in a more coordinated manner for sustainable development.
■ ***Build resilience***. Climate variability and change are threatening and providing a multiplier effect on existing challenges to the SADC’s development agenda. The region is a climate change hot spot; this is particularly disconcerting due to dependence on climate sensitive sectors of agriculture and energy. Already increasing frequency and intensity of extreme weather events (droughts and floods) has retarded economic growth and set back regional targets. The WEF nexus approach provides opportunity for building regional resilience to climate change and mitigating vulnerabilities through coordinated WEF infrastructure development, improved management of transboundary natural resources, maximising on regional comparative advantages for agricultural production, and unlocking more resources for climate proofing through increased efficiencies. Overall, implementing the WEF nexus would promote sustainable development, and this particular recommendation on resilience building is aligned to SDG 13 on climate action.
■ ***Promote investment in infrastructure development***. The WEF nexus approach promotes investment in resource efficient technologies as well as innovative policies and institutional support to decouple intensive resource use from food production. Once effective strategies are in place, investment is attracted that would benefit the use of modern technologies that include production and use of renewable energy through hydropower, solar-powered water pumps for irrigation, generation of electricity from crop residues, production of biogas from manure, and introduction of trees or perennials on farms to produce wood for on-farm energy purposes among others. A regional WEF nexus approach would lead to infrastructure development in countries that currently lag. For example, while northern parts of SADC have abundant water resources, they face economic water scarcity due to lack of infrastructure for storage and distribution. A regional WEF nexus approach could lead to investment in dam construction, hydropower, and water distribution infrastructure in these states. For example, the Lesotho Highlands Water Project has boosted infrastructure development, provided employment, hydropower and revenue in Lesotho whilst addressing South Africa’s water scarcity. Similarly, WEF-driven innovative infrastructure has the potential to boost economic growth with new opportunities for SADC to increase its profile as a global production centre.

## 7. Conclusions

The water-energy-food nexus draws on holistic, socio-ecological systems perspective that recognise the value of all sectors in equal terms. Climate determines water availability, potential agricultural production and energy availability, particularly in areas dependent on hydropower. Climate variability and change is the main cause of the fluctuations in water availability as well as access to energy and food resources, triggering trade-offs across the whole WEF nexus. Southern Africa is highly exposed to climate variability and change due to the high dependence on climate sensitive sectors of water and agriculture and reliance on hydropower for energy. These sectors are the mainstay of the economies of regional countries, yet there is strong evidence of the effects of individual climate extremes. Climate change is a cross-cutting challenge that needs to be tackled by more than one climate sensitive sector. The integration of climate change adaptation strategies into the water-energy-food nexus offers opportunities to create proper resource coordination, harmonise activities across all sectors, improve resilience, and reduce vulnerabilities to attain regional development targets. Resource scarcity is one of the primary constraints for individual sectors to meet the ambitions of the Sustainable Development Goals, and in general the development aspirations of Southern African Development Community. The water-energy-food nexus presents an opportunity for policymakers, researchers, and development agencies to integrate the sectors to optimise the use of the resource base, maximise synergies, and minimise trade-offs, and has grown to be an essential tool to achieve the Sustainable Development Goals on poverty alleviation, zero hunger, provision of water and sanitation, and access to affordable and reliable energy (Goals 1, 2, 6, and 7, respectively). There are also significant implications for health and sanitation as higher temperatures and lower rainfall are projected in the region, which may result in mal-nutrition and disease outbreaks and prevalence. The water-energy-food nexus offers opportunities to integrate and manage these challenges in a more sustainable manner and promote regional peace and cooperation, harmonisation of legislation, policies, and strategies in a region of transboundary resources like southern Africa. Sectoral policies that are not linked to each other are the cause of unsustainability and unbalanced resource development. However, successful implementation of the nexus at regional level requires political commitment, supported by technological innovations that allow producing more with less resources.
